# Macropinocytosis dependent entry of Chikungunya virus into human muscle cells

**DOI:** 10.1371/journal.pntd.0007610

**Published:** 2019-08-26

**Authors:** Ching Hua, Regina Lee, Khairunnisa Mohamed Hussain, Justin Jang Hann Chu

**Affiliations:** 1 Laboratory of Molecular RNA Virology and Antiviral Strategies, Department of Microbiology and Immunology, Yong Loo Lin School of Medicine, National University of Singapore, Singapore; 2 Collaborative and Translational Unit for HFMD Singapore, Singapore, Institute of Molecular and Cell Biology, Agency for Science, Technology and Research (A*STAR), Singapore, Singapore; International Centre for Genetic Engineering and Biotechnology, INDIA

## Abstract

Chikungunya virus (CHIKV) is a re-emerging arbovirus known to cause chronic myalgia and arthralgia with high morbidity. CHIKV is now considered endemic in many countries across Asia and Africa. In this study, the susceptibility of various human, mammalian and mosquito cell lines to CHIKV infection was evaluated. CHIKV infection was found to be cell-type dependent and virus strain-specific. Furthermore, SJCRH30 (human rhabdomyosarcoma cell line) was showed to be highly permissive to CHIKV infection, with maximum production of infectious virions observed at 12 h.p.i. Pre-infection treatment of SJCRH30 with various inhibitors of endocytosis, including monodansylcadaverine (receptor-mediated endocytic inhibitor), dynasore (clathrin-mediated endocytic inhibitor), as well as filipin (caveolin-mediated endocytosis inhibitor), resulted in minimal inhibition of CHIKV infection. In contrast, dose-dependent inhibition of CHIKV infection was observed with the treatment of macropinocytosis inhibitor, 5-(N-ethyl-N-isopropyl)amiloride (EIPA). Furthermore, siRNA-mediated knockdown of sortin nexin 9 (SNX9) a protein involved in macropinosome formation, also resulted in a significant dose-dependent reduction in viral titre. By performing a virus entry assay, CHIKV particles were also observed to colocalize with FITC-dextran, a macropinosome marker. This study shows for the first time, that the infectious entry of CHIKV into human muscle cells is mediated by macropinocytosis. Together, the data from this study may pave the way for the development of specific inhibitors that target the entry process of CHIKV into cells.

## Author summary

This study revealed the differences in susceptibility of various human, mammalian and mosquito cell lines to CHIKV infection. CHIKV infection was found to be cell-type dependent and virus-strain specific. Additionally, two human muscle cell lines, SJCRH30 (rhabdomyosarcoma cell line) and HSMM (human skeletal muscle myoblasts), were shown to be highly susceptible to infection by different CHIKV strains. Pre-infection treatment of SJCRH30 and HSMM with a macropinocytosis inhibitor, 5-(N-ethyl-N-isopropyl)amiloride (EIPA) showed a dose-dependent inhibition. Additionally, knockdown of a protein involved in macropinocytosis formation, SNX9, revealed that CHIKV infection of SJCRH30 cells relies on macropinocytosis. Results were confirmed with a FITC-dextran assay, which showed colocalisation between CHIKV particles and macropinosomes during viral entry. Overall, this study may contribute to the development of therapeutic interventions using specific inhibitors that target the entry of CHIKV into muscle cells.

## Introduction

Chikungunya virus (CHIKV) is an arthropod-borne virus belonging to genus *Alphavirus* and family *Togaviridae*. CHIKV is most commonly transmitted by *Aedes* (*Ae*.) mosquitoes. First isolated in 1952 in Tanzania, CHIKV transmission begins with infection of female mosquitoes via a viremic blood-meal taken from a susceptible vertebrate host and subsequent transmission to another vertebrate host [1]. CHIKV-infected individuals may suffer from persistent polyarthralgia and myalgia, often resulting in significant dips in their quality of life. Despite the disease having impacted millions around the world in the last two decades, no antiviral against CHIKV infection is available. CHIKV treatment remains primarily supportive, with key areas of focus being management of clinical symptoms and prevention of vector transmission. It is therefore imperative to gain a deeper understanding of the pathogenesis of CHIKV infection, in order to drive the development of effective antiviral therapeutics.

The alphaviral entry process has been previously reported to be dependent on clathrin-mediated endocytosis. This is followed by acidification of the endosomes, which triggers fusion of the viral envelope with the endosomal membrane, releasing the viral genome for translation and replication [2]. Nevertheless, the ability of alphaviruses to enter host cells via alternative mechanisms has also been reported [3]. An early study on Sindbis virus (SINV) entry showed the translation of viral RNA in the cytosol even upon treatment with chloroquine and ammonium chloride, suggesting that the acidification of endosomes is not necessary essential for infection to occur [4]. A recent study also reported the progression of SINV infection in various cell lines even in the absence of low pH-induced endocytosis, indicative of clathrin-independent entry [5,6]. For instance, upon siRNA knockdown of the clathrin heavy chain, CHIKV infection of both HEK293 and HeLa cells was found to be unaffected [7]. Mutants of Eps15 and Rab5, as well as endocytic inhibitors only resulted in partial block in CHIKV infection, supporting the hypothesis that several pathways are hijacked by CHIKV to facilitate its entry into target cells [7]. These clathrin-independent pathways could include caveolae-dependent endocytosis, macropinocytosis or dynamin-dependent clathrin- and caveolin-independent endocytosis. The caveolar/lipid raft pathway transports internalised virus to neutral-pH caveosomes to be sorted, before redistribution and delivery of the cargo to smooth endoplasmic reticulum (SER), usually through microtubule-directed movements [8]. Viruses known to commonly utilize this pathway include SV40, enterovirus 1, filoviruses and polyomaviruses [8].

Macropinocytosis is different from other pinocytic pathways. After virus binding to the cell membrane, it activates the receptor tyrosine kinases (RTKs) or other signaling molecules to further activate the intracellular multi-branched signaling cascades. Thereafter, actin rearrangement will be triggered, causing the appearance of numerous irregular ruffles and blebs on the cell membrane observed. These ruffles will then collapsed towards the cell membrane, enveloping the virus-receptor complex together with the dissociated virus and other fluid-phase macromolecules [9–13]. The process is triggered upon cell stimulation to internalize large amounts of fluids or solutes into large uncoated vesicles sized between 0.5 to 10 μm called macropinosomes [13,14]. Activation of macropinocytosis occurs when a virus binds to a co-receptor, which results in signal transduction to host cells via cellular proteins such as phosphatidylinositol 3-kinase (PI3K), protein kinase C and the Rho GTPase Rac1 [11,15]. Dependent on actin polymerization, macropinosomes are then formed by closure of lamellipodia or membrane ruffles [16]. Viruses known to enter via this pathway include vaccinia virus [11] and adenovirus 2 [17].

Several studies have demonstrated susceptibility of various cell lines to CHIKV infection, with the rapid induction of apoptosis observed [1,18]. Epithelial cells, endothelial cells, primary fibroblasts and monocyte-derived macrophages have been found to be permissive to infection [1,18]. Skeletal muscle progenitor cells or satellite cells have also found to co-stain with CHIKV antigens, indicating their permissiveness to CHIKV replication, which may explain the recurrent myalgia experienced by infected individuals [19]. In addition, *in vivo* murine studies suggest fibroblasts as the primary cellular target for CHIKV infection, confirming earlier *in vitro* findings and also accounting for CHIKV arthralgia and myalgia observed in patients [20]. Consistent with reports of neurological involvement, neurons and glial cells are also observed to be susceptible to CHIKV infection [21]. In a macaque model, persistent infection of liver tissues, as well as significant levels of hepatocyte cell death indicated the involvement of hepatocytes in CHIKV pathogenesis [22]. Determining the cell types to which CHIKV can attach and productively infect is crucial in understanding the pathogenesis and pathophysiology of CHIKV infection in humans. This is essential in the development of effective therapeutics against CHIKV infection.

In this study, the susceptibility of a panel of mammalian and arthropod cell lines to infection with three strains of CHIKV was evaluated. A number of highly permissive cell lines were indentified, including SJCRH30, a human rhabdomyosarcoma cell line. Treatment with a variety of endocytosis inhibitors revealed the possible involvement of macropinocytosis during CHIKV entry in SJCRH30 and HSMM (primary skeletal human myoblasts). This was further confirmed by the siRNA-mediated knockdown of SNX9 as well as a FITC-dextran assay in SJCRH30 cells. This study reveals the possible involvement of macropinocytosis in the CHIKV entry of skeletal muscle cells, indicating that macropinocytosis is a potential therapeutic target for the development of antivirals against CHIKV.

## Materials and methods

### Cell culture

Eighteen different cell lines were used in this study (Table 1), which included, HBMEC (ScienCell); SK-N-SH (ATCC HTB-11); HaCaT (ATCC PCS-200-011); Rhadomyosarcoma (ATCC CCL-136); SJCRH30 (ATCC CRL-2061); A549 (ATCC CCL-185); HUH 7 (Dr Priscilla Yang, Harvard Medical School, USA); HUH 7.5 (Dr Yoichi Suzuki, Osaka Medical College Hospital, Japan); HepG2 (ATCC HB-8065); HEK293A (Life Technologies); HEK293T (Life Technologies); HeLa CCL2 (ATCC CRM-CCL-2); Vero (ATCC CCL-81); Vero C1008 (ATCC CRL-1586); Baby Hamster Kidney (BHK) 21 (ATCC CCL-10); Chinese Hamster Ovary (CHO) (ATCC CCL-61); C2C12 (ATCC CRL-1772) and *Aedes*. *albopictus* C6/36 (ATCC CRL-1660). The growth medium used are from Sigma-Aldrich Corp., St Louis, MO, USA. Cell lines 1 to 17 (Table 1) were cultured in RPMI and DMEM 10% fetal bovine serum (FBS) (Capricorn Scientific, South America), at 37°C in the presence of 5% CO_2_. In addition, C6/36 cells were cultured in L-15 media supplemented with 10% heat-inactivated FBS (Capricorn Scientific, South America) at 28°C. HSMM cells were cultured in skeletal muscle cell growth medium (SkGM) (Lonza) supplemented with growth factors and 10% FBS (Lonza) at 37°C with 5% CO2.

**Table 1:** Susceptibility of cell lines with different strains of CHIKV. Values were scored based on differences in viral titre observed upon comparison of peak viral titres to 0h.

Tissues				
	Cell Lines	CHIKV 122508	CHIKV 6708	CHIKV 0708
**HUMAN**				
**Brain**	**HBMEC**	**+**	**-**	**-**
	**SK-N-SH**	**+++**	**+++**	**+++**
**Keratinocytes**	**HaCaT**	**-**	**-**	**-**
**Muscle**	**Rhabdomyosarcoma**	**+++**	**-**	**-**
	**SJCRH30**	**+++**	**+++**	**+++**
**Lungs**	**A549**	**-**	**-**	**-**
**Liver**	**Huh 7**	**+++**	**-**	**-**
	**Huh 7.5**	**+++**	**-**	**+**
	**HepG2**	**+++**	**+**	**-**
**Kidney**	**HEK293A**	**+++**	**+++**	**-**
	**HEK293T**	**+++**	**+**	**-**
**Cervical**	**HeLa CCL2**	**+++**	**+++**	**+++**
**NON-HUMAN PRIMATES**				
**Kidney**	**Vero**	**+++**	**+**	**+**
	**Vero C1008**	**+++**	**+++**	**+++**
**MURINE**				
**Muscle**	**C2C12**	**+++**	**+++**	**+++**
**Kidney**	**BHK-21**	**+++**	**+++**	**+++**
**Ovary**	**CHO**	**+++**	**+**	**-**
**ARTHOPOD**				
**Mosquito**	**C6/36**	**+++**	**+++**	**+++**

(-)–No difference compared to 0h; (+) - 1 to 2 log_10_ (PFU/ml) increase; (++) 2 to 3log_10_ (PFU/ml) increase; (+++) - 3-log_10_ (PFU/ml) increase

### CHIKV infection

CHIKV-0708 (Singapore/07/2008 strain, kindly provided by National Public Health Laboratory, Ministry of Health, Singapore) was propagated in BHK-21 cells. BHK-21 cells were infected with CHIKV-0708 and incubated for 4 days at 37°C with 5% CO_2_ prior to harvesting of viral supernatants and storage at -80°C. CHIKV-6708 (LK(EH)CH6708, Accession No.: EU441882.1) and CHIKV-122508 (SGEHICHID122508, Accession No.: FJ445502.2) were both obtained from Environmental Health Institute (EHI), National Environment Agency (NEA) (Singapore). CHIKV-6708 and CHIKV-122508 were propagated in C6/36 cells and incubated for 4 days at 28°C prior to harvesting of viral supernatants and storage at -80°C. All CHIKV strains are of ECSA lineage and were previously isolated from individual patients during the Singapore CHIKV outbreak in 2008.

### Viral plaque assay

For quantification of viral titres, BHK-21 cells were seeded on 24-well plates and incubated for 24h. Viral supernatants were subjected to ten-fold serial dilutions before infection of BHK-21 monolayers for 1.5h at 37°C with 5% CO_2_ to allow virus infection. Phosphate-buffered saline (PBS) was then used to wash off unbound viruses after incubation. An overlay medium containing 1% Aquacide II (Calbiochem) in RPMI supplemented with 2% FBS was added into each well before incubation for three days at 37°C with 5% CO_2_. Plaques were formed when individual cell are infected and subsequently lysed forming a plaque. The overlay medium was then removed and cells were fixed and stained using a solution containing 3.2% paraformaldehyde and 0.2% crystal violet was used to visualize plaques. Viral titres were expressed in log_10_ plaque-forming unit per millilitre (PFU/mL) [23].

### Establishment of viral growth kinetics

Cells were seeded (1 x 10^5^ cells per well) in 24-well plates and infected with different strains of CHIKV at a multiplicity of infection (M.O.I) of 10 for 1.5 hrs at 37°C with 5% CO_2_. Infected cells were then washed three times with PBS and overlaid with 1mL of maintenance media (RPMI or DMEM with 2% FBS) per well. Supernatants were collected every 6 hrs for the first 24 hrs, and subsequently daily for 5 days, with the first time-point (time 0) collected immediately after the addition of the maintenance media to account for leftover unbound virus. Infectious virus titres of the supernatants collected were determined by viral plaque assay.

### Immunofluorescence assay (IFA)

Cells were seeded onto coverslips in 24-well plates and incubated overnight to achieve 75–80% confluency. For IFA, cells were seeded at the following densities: 4×10^4^ cells/well for SJCRH30, 8×10^4^ cells/well for SK-N-SH, 7×10^4^ cells/well for HeLa CCL2, 4×10^4^ cells/ml, 7×10^4^ cells/ml of C2C12 and 2x10^5^ cells/well for C6/36. Cell monolayers were infected with CHIKV-0708 at a multiplicity of Infection (MOI) of 10 for 1 hour at 37°C to allow viral attachment and entry. Infected cells were washed twice with PBS and topped up with maintenance media to allow virus growth. Cells were then incubated for 24 h.p.i before fixation. Supernatants were removed and cells were washed once with PBS prior to fixation in ice-cold methanol for 15 min. This was followed by three washes of cold PBS prior to incubation with primary antibodies. The cells were then subjected to immunofluorescence staining using appropriate antibodies and/or fluorescent dyes, such as primary anti-alphavirus mouse monoclonal antibodies (Santa Cruz Biotechnology, 1:100 dilution), followed by secondary goat anti-mouse IgG conjugated to fluorescein isothiocyanate (FITC; Invitrogen, 1:500 dilution) antibody. Cell nuclei were counter-stained with DAPI (Sigma Aldrich, Duolink *in situ* mounting media). Coverslips were then viewed at DAPI and FITC channels at 100× magnification (Olympus IX81, inverted microscope) [24].

### Inhibitory studies

Compounds used in this study, namely, dynasore (inhibitor of clathrin-mediated endocytosis), filipin (inhibitor of caveolae-dependent endocytosis), monodansylcadaverine (inhibitor of receptor-mediated endocytosis) and 5-(N-ethyl-N-isopropylamiloride (EIPA) (inhibitor of macropinocytosis were dissolved in dimethyl sulfoxide (DMSO) according to manufacturer recommendations to prepare master-stocks for storage. Dynasore: 0.1, 0.5, 1, 5, 10, 50, 100, 200 μM, filipin: 0.5, 1, 1.5, 2, 3 μg/mL, monodansylcadaverine 50, 100, 150, 200, 250, 500 μM and EIPA: 10, 50, 150, 200 μM. All compounds were obtained from Sigma Aldrich.

Cells were seeded into 24-well plates and incubated overnight prior to drug inhibitory assays. Pre-infection treatment drug assays were performed in order to ensure that identify potential inhibitors of CHIKV entry processes. Compunds were diluted to relevant working concentrations (dynasore: 0.1, 0.5, 1, 5, 10, 50, 100, 200 μM, filipin: 0.5, 1, 1.5, 2, 3 μg/mL, monodansylcadaverine 50, 100, 150, 200, 250, 500 μM and EIPA: 10, 50, 150, 200 μM) in maintenance media and incubated with cell monolayers for 3 hours at 37°C. The inhibitors were then removed, and cell monolayers were washed twice with PBS prior to CHIKV-0708 infection. After 1.5 hrs of CHIKV-0708 infection at an M.O.I. of 10, the cells were washed thrice with PBS, replaced with fresh maintenance media and incubated for 24h before harvesting viral supernatants for titration via plaque assays. Three independent experiments were carried out for each inhibitor used [25].

### Cell viability assay

Cells were seeded in 96-well plates and incubated overnight at 37°C with 5% CO_2_. Upon treatment with respective inhibitors for 3 hrs or siRNAs for 48 hrs, 10μL of alamarBlue reagent (Invitrogen) was added to each well, before incubation at 37°C with 5% CO_2_ for 4 hrs. Fluorescence was detected at an excitation wavelength of 570 nm and an emission wavelength of 585 nm on an Infinite 200 Pro multiplate reader (Tecan). Cell viability was analysed by normalizing to untreated cells [26].

### SDS-PAGE and western blot

Total cell lysate of SJCRH30 was harvested at 48 hours post-transfection by incubating cell monolayers with 1% sodium dodecyl sulfate (SDS) for 10 minutes at 4°C before subjecting to centrifugation at 16,000 ×*g* at 4°C for 30 minutes to pellet cell debris. Protein supernatants were stored at -20°C. Prior to SDS-PAGE, protein samples were quantified using Bradford assay (Bio-Rad Dye Reagent Concentrate). Equal amount of proteins were loaded into a 10% SDS-polyacrylamide gel and resolved at constant voltage of 100 V for 3 hours. The proteins were then transferred onto a nitro-cellulose membrane (Bio-Rad) electrophorectically using Trans-Blot Turbo Transfer System (BioRad) at 17 V for 7 minutes. Blocking of non-specific proteins was carried out using 5% bovine serum albumin (BSA) in tris-buffered saline with Tween-20 (TBST) at room temperature for 2 hours. The membrane was then probed with mouse anti-human SNX9 monoclonal antibody (Santa Cruz Biotechnology, 200 μg/ml, 1:100 dilution) and mouse anti-human ß-actin (Millipore, 0.05mg/ml, 1:10,000 dilution), respectively, overnight at 4°C before. Blots were washed thrice with TBST and incubated with horseradish peroxidase-conjugated anti-mouse IgG (Calbiochem, 1:10,00 dilution) for 1 hour at room temperature. Protein bands were developed by SuperSignal West Pico Chemiluminescent Substrate (Thermo Scientific). The bands were detected by C-digit Blot scanner (*Li-Cor*). Band intensities were quantified using the LI-COR Image Studio programme. Protein bands were normalised against ß-actin which served as a loading control and compared against non-targeting siRNAs across the concentrations tested [27].

### siRNA transfection

siRNA targeting SNX9 was selected to further evaluate the role of macropinocytosis in CHIKV-0708 infected of SJCRH30. Specific gene-targeting SNX9 siRNA was dissolved in DEPC-treated reverse osmosis water to a final stock concentration of 100mM and incubated at room temperature for 30 mins with gentle agitation. SNX9 siRNA was diluted to desired working concentrations with serum-free media (Lonza) and transfection reagent (Dharmafect-1, Dharmacon). The specific individual siRNA that were directed against each of the respective genes were then transfected into SJCRH30. At 48 hours post-transfection, cells were infected with CHIKV-0708. Viral supernatants were then harvested at 24 h.p.i for plaque assays.

### FITC-dextran assay

SJCRH30 cells (4×10^4^ cells/well) were seeded on 24-well plate with coverslips and incubated overnight. SJCRH30 were starved with serum-free medium for 24 h. Cells were then put into 4°C for 15 mins before subjecting to CHIKV-0708 infection. SJCRH30 cells were then infected at M.O.I of 10 for 30 mins at 4°C to facilitate virus binding. Cell monolayers were then shifted to 37°C for 10 mins to allow viral entry. Alongside, serum-starved SJCRH30 cells were also pre-treated with EIPA and 0.1% DMSO for 2 h. Cells were then washed once with PBS and shifted to 4°C for 15 mins before subjecting to CHIKV-0708 infection. Similarly, SJCRH30 cells were then infected at M.O.I of 10 for 30 mins at 4°C to facilitate virus binding. Cell monolayers were then shifted to 37°C for 10 mins to allow viral entry. Fluorescein Isothiocyanate-Dextran 70kDa (FITC-Dextran, Sigma Aldrich, 1mg/mL) was added in the dark and plates were incubated for 30 mins at 37°C, and washed thrice with PBS. Cells were then fixed with methanol for 10 mins at -20°C and washed thrice with PBS. Subsequently, cell monolayers were stained with a polyclonal rabbit anti-CHIKV E2 antibody (in-house production, 1:100 dilution) for 1 hour at 37°C, followed by secondary goat anti-rabbit TRITC 594 antibody (Thermoscientific, USA, 1:300 dilution) for 1h hour at 37°C. Additionally, cell nuclei were counter-stained with DAPI (Sigma Aldrich, Duolink *in situ* mounting media). Stained cells were then viewed at DAPI, FITC-Dextran and TRITC-594 channels at 100× magnification (Olympus IX81, inverted microscope) [28].

### Statistical analysis

Where applicable, statistical analyses were performed repeatedly using one-way ANOVA with Dunnett’s post-test. The significance level was set at p < 0.05 (*), p < 0.01 (**) or p < 0.001 (***). Data shown throughout the study is presented as means from three independent experiments.

## Results

### Susceptibility profiles of various cell lines to CHIKV infection

The growth kinetics of various CHIKV strains isolated from patients in Singapore were evaluated in eighteen cell lines. Three strains belonging to the ECSA lineage, namely, CHIKV-0708, CHIKV-122508 and CHIKV-6708 were used to infect a total of twelve human cell lines, five murine cell lines and one arthropod cell line as shown in Table 1. All infections were carried out at a multiplicity of infection (M.O.I) of 10.

**Fig 1:**
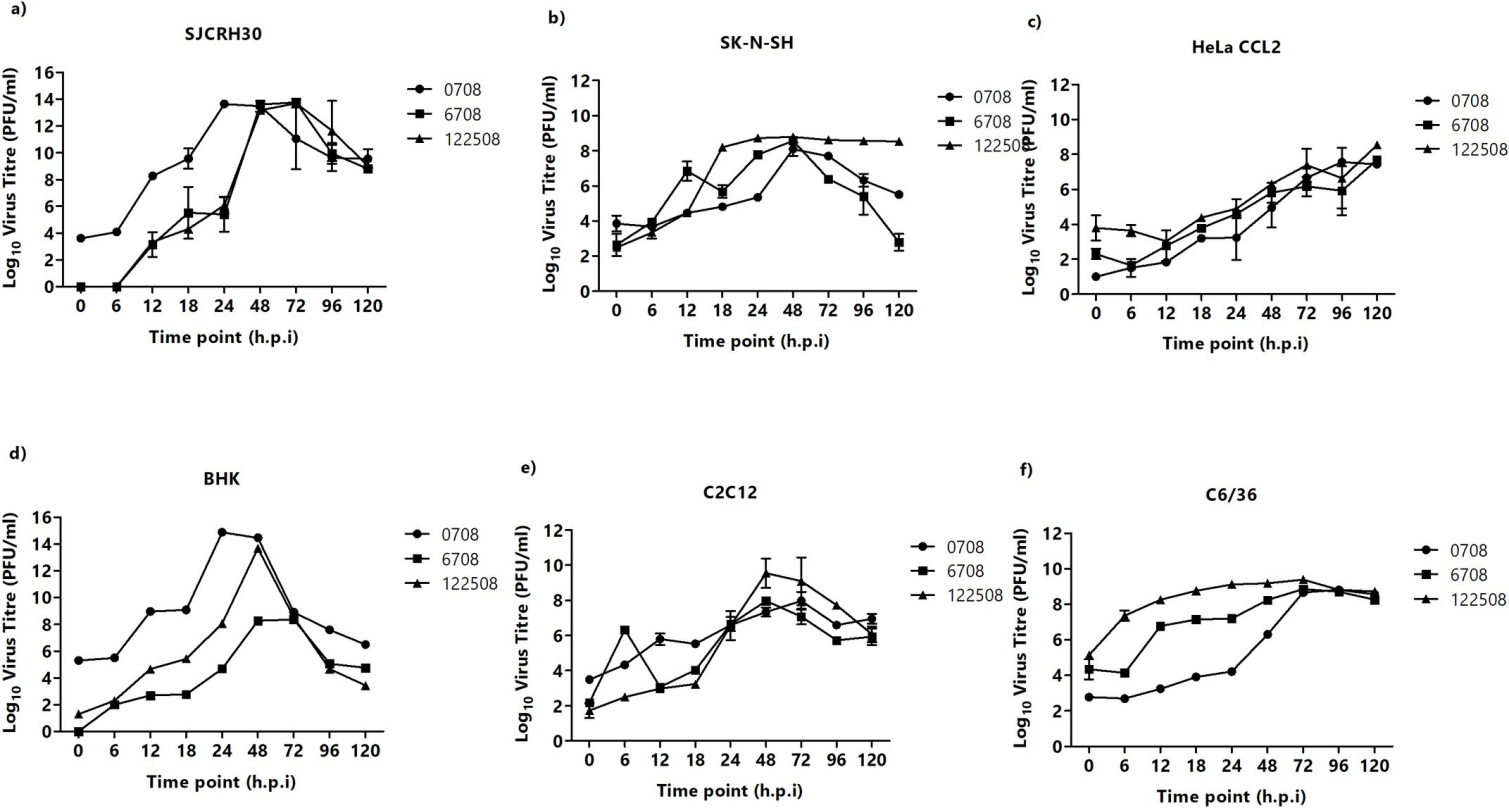
Susceptibility of six different cell lines infected with three Chikungunya strains–CHIKV-122508, CHIKV-6708 and CHIKV-0708. Cells were infected with different CHIKV strains across various timepoints. (A) SJCRH30, (B) SK-N-SH, (C) HeLa CCL2, (D) BHK-21, (E) C2C12 and (F) C6/36. CHIKV strains CHIKV-0708, CHIKV-6708 and CHIKV-122508 are represented by (black circle), (black square) and (black triangle).

Among the various human cell lines tested, SJCRH30 was found to be the most susceptible to all the three CHIKV strains tested (Fig 1A).

As observed in Fig 1A, the infectious titres of all CHIKV strains were progressively increased, although the time-point for peak titres of CHIKV-0708 (10^8^ to 10^14^ PFU/mL) occurred between 12 h.p.i to 24 h.p.i., while those of CHIKV-122508 and CHIKV-6708 occurred at 48 h.p.i. Similar growth trends were observed for all three CHIKV strains in SK-N-SH cells, with the infectious titre peaking at 48 h.p.i. (Fig 1B). In HeLa CCL2 cells, a gradual increase in virus titre was observed, with a peak titre of 10^6^ PFU/mL at 48 h.p.i (Fig 1C).

Both murine cell lines, BHK-21 and C2C12, were observed to be highly susceptible to all CHIKV strains. Infected C2C12 cells showed peak infectious titres at 48 h.p.i. for CHIKV-6708 and CHIKV-122508, respectively (10^8^ and 10^10^ PFU/mL) (Fig 1E), and 10^7^ PFU/mL for CHIKV-0708 at 24 h.p.i. In BHK-21 cells, more than 10^14^ PFU/mL was observed at 24 h.p.i. for CHIKV-0708 (Fig 1D). Similarly, high viral titres were seen for CHIKV-122508 and CHIKV-6708, albeit at a later infection time-point of 48 h.p.i. in BHK-21 cells (Fig 1D).

In C6/36 cells, progressively increasing growth curves could be observed for all CHIKV strains, with peak infectious titres occurring at different time-points. CHIKV-0708 and CHIKV-6708 titres peaked at 72 h.p.i., while CHIKV-122508 showed high infectious titre of 10^8^ PFU/mL from 6 h.p.i onwards (Fig 1F). The six cell lines shown in Fig 1 are highly susceptible to all three CHIKV strains and exhibit strain-specific differences in time-points and magnitude of peak infectious viral titres.

In contrast, the remaining twelve cell lines tested displayed relatively lower levels of permissiveness to CHIKV, with some cell lines being unsupportive of CHIKV replication and others showing lower magnitudes of peak titres (Table 1 and S1 Fig). The differences in permissiveness to CHIKV infection is in agreement with previous studies and is likely to be related to the *in vivo* tropism of CHIKV [1,18]. Values were scored based on differences in viral titre observed upon comparison of peak viral titres to 0h. Cell lines with no difference throughout the 5 days of infection were scored as (-). Cell lines that showed a 1 to 2 log_10_ (PFU/ml) increase in virus titre were scored as (+), while cell lines with an increase of 2 to 3log_10_ (PFU/ml) were scored as (++) and lastly, cell lines with an increase of 3-log_10_ (PFU/ml) or higher were scored as (+++).

**Fig 2:**
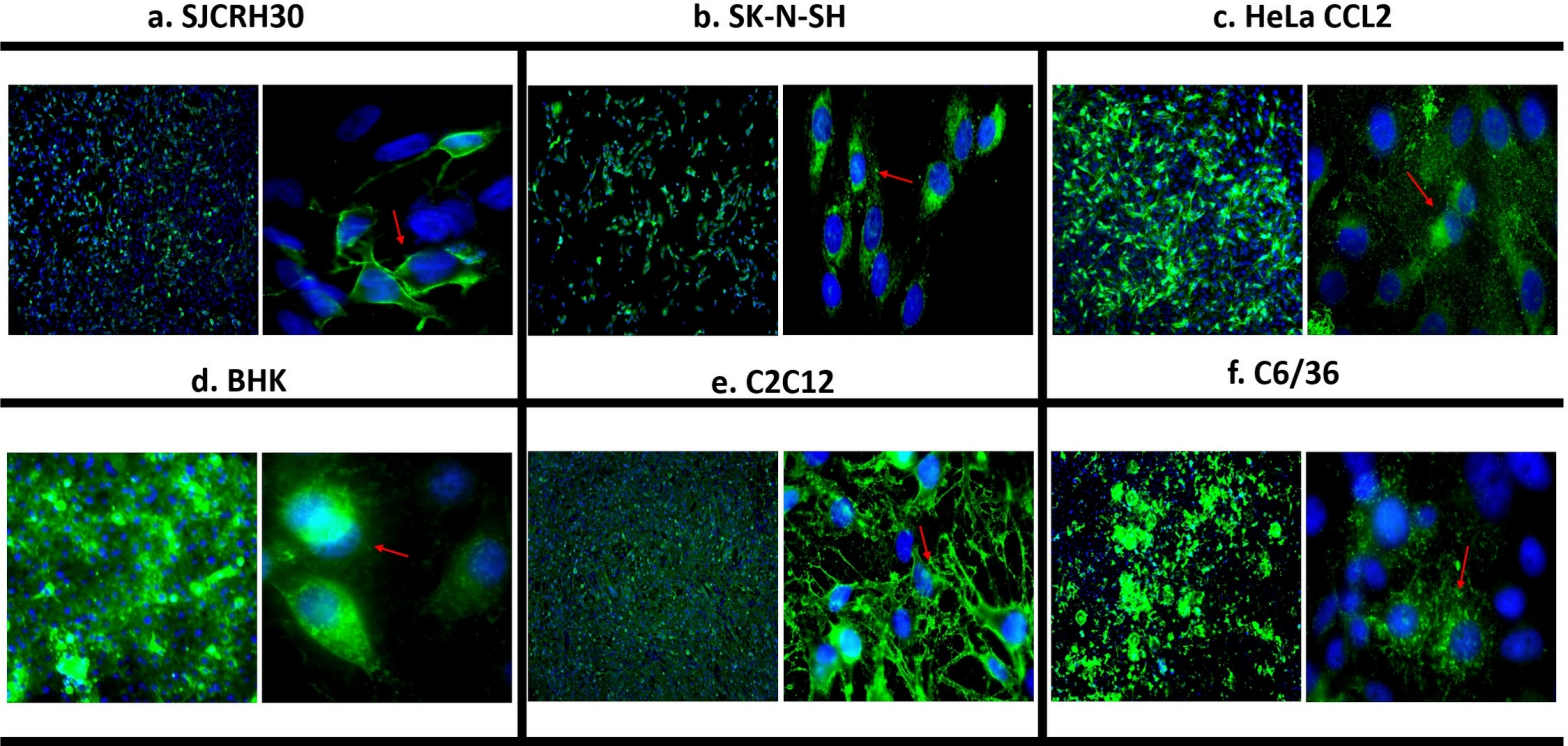
Immunofluorescence assay analysis of top six susceptible cell lines. (A) SJCRH30, (B) SK-N-SH, (C) HeLa CCL2, (D) BHK-21, (E) C2C12 and (F) C6/36. Cells infected with CHIKV-0708 were fixed and stained at 24 h.p.i. High infection rates were observed, as depicted by the green fluorescent signal. CHIKV viral proteins were also observed to accumulate within the cytoplasm and towards the plasma membrane, as well as the perinuclear regions (red arrowheads). Cell nuclei were counterstained with DAPI (blue).

The expression of viral antigen in the six highly permissive cell lines (SJCRH30, SK-N-SH, HeLa CCL2, BHK-21, C2C12 and C6/36) was further analyzed by IFA. At 24 h.p.i, majority of the cells were found to be positive for CHIKV antigen (envelope protein), which accumulated within the cytoplasm and along the plasma membrane, as indicated by the red arrows in Fig 2.

### CHIKV enters muscle cells primarily via macropinocytosis

Given the major involvement of muscle cells in CHIKV pathogenesis *in vivo*, as well as the high permissiveness of SJCRH30 cells to CHIKV in our study, SJCRH30 cells were selected for downstream studies to decipher the entry process of CHIKV. In addition, HSMM cells, a primary human skeletal muscle myoblast line known to be infected by CHIKV *in vitro* was also included for greater relevance to clinical settings [29]. All downstream experiments were carried out with CHIKV-0708, given its ability to develop high peak titres in SJCRH30 cells (Fig 1A) and HSMM cells [29] by 24 h.p.i.

**Fig 3:**
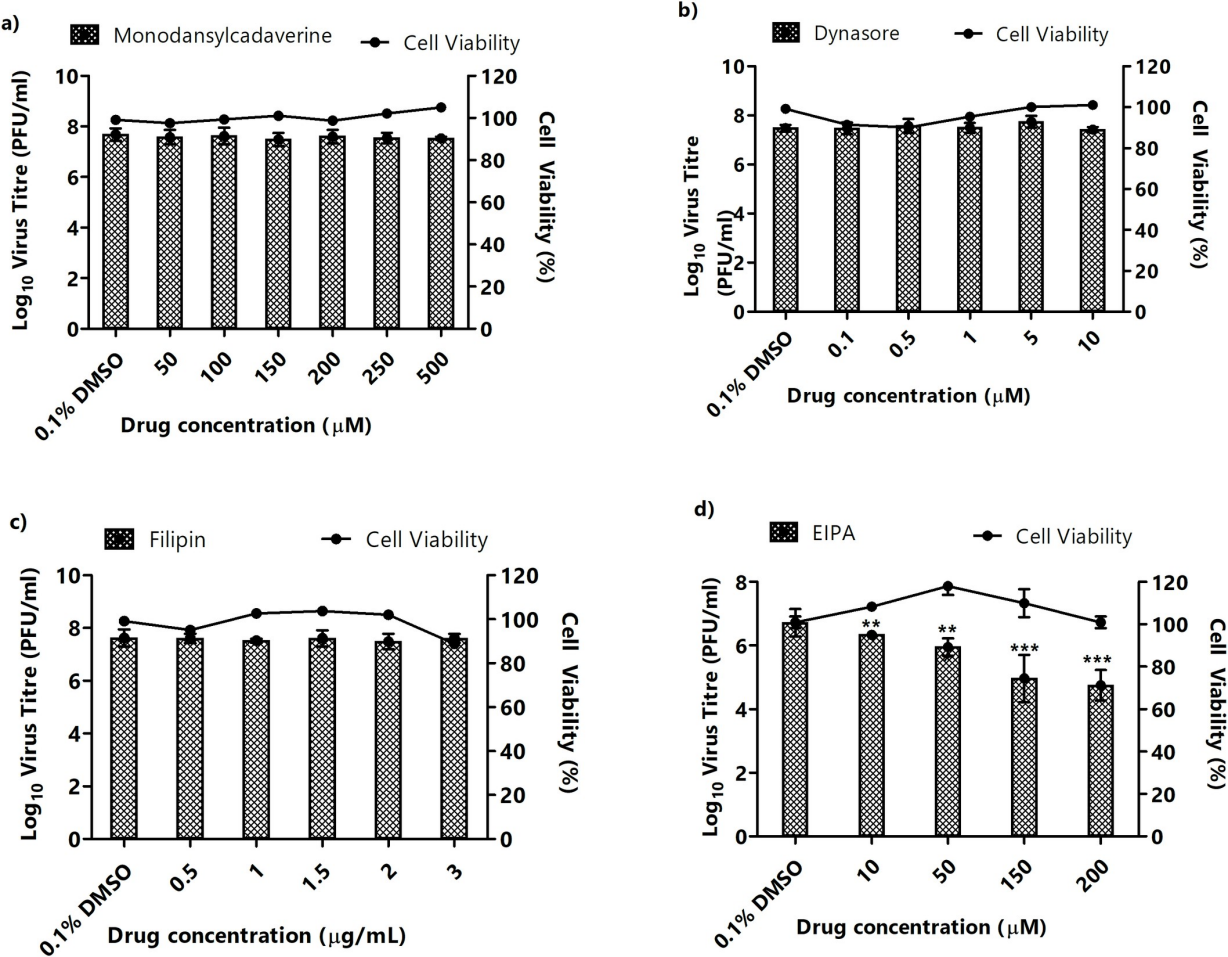
Pre-infection treatment assays of SJCRH30 with inhibitors targeting different entry pathways. SJCRH30 were pre-treated with different inhibitors for 3 hours before CHIKV infection. Supernatants were harvested at 24 h.p.i for viral plaque assays. The virus titre expressed as log_10_ PFU/mL is plotted against the concentrations of inhibitor used. Significant dose-dependent inhibition of CHIKV infection was observed with (D) EIPA-treated cells. In contrast, no inhibition was observed in (A) monodansylcadaverine-, (B) dynasore- and (C) filipin-treated cells. Cell viability upon drug treatments is represented by the line graphs. The asterisk indicates *p values <0.05, **p values of <0.01 and ***p values <0.001.

Virus entry occurs via several endocytic pathways, with the most common being clathrin- and caveolae-mediated endocytosis [30]. To further investigate the specific entry pathway(s) utilized by CHIKV in muscle cells, inhibition studies using various inhibitors of endocytosis were conducted. The inhibitors were used to treat SJCRH30 and HSMM prior to infection with CHIKV-0708. Minimal cellular toxicity was observed across the various concentrations of all inhibitors tested in this study (Fig 3).

**Fig 4:**
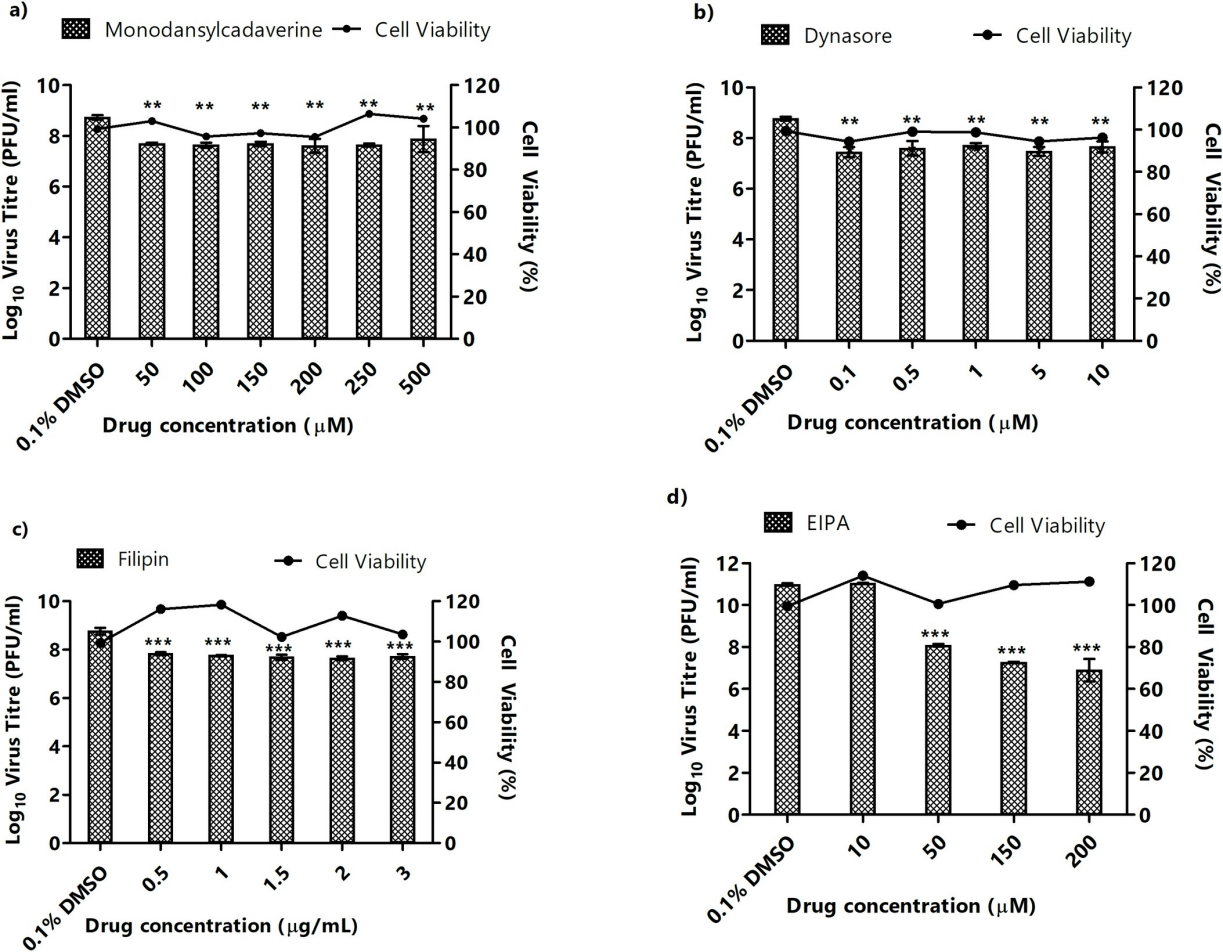
Pre-infection treatment assays of HSMM with inhibitors affecting different entry pathways. HSMM were pre-treated with different inhibitors for 3 hours before CHIKV infection. Supernatants were harvested at 24 h.p.i for viral plaque assays. The virus titre expressed as log_10_ PFU/mL is plotted against the concentrations of inhibitor used. Significant dose-dependent inhibition of CHIKV infection was observed with (d) EIPA-treated cells. In contrast, significant but non-dose-dependent inhibition was observed in CHIKV entry into (A) monodansylcadaverine-, (B) dynasore- and (C) filipin-treated cells is observed. Cell viability upon treatment with inhibitors is represented by the line graphs. The asterisk indicates *p values <0.05, **p values of <0.01 and ***p values <0.0.01.

Pre-infection treatment of cells with monodansylcadvarine, a specific inhibitor of receptor-mediated endocytosis [31], resulted in minimal inhibition of CHIKV infection in SJCRH30 (Fig 3A), while pre-infection treatment in HSMM induced a significant albeit non-dose dependent decrease of approximately 1 log_10_ PFU/mL of CHIKV titre (Fig 4A).

Dynasore, a GTPase inhibitor that targets dynamin and blocks endocytosis, particularly clathrin-mediated endocytosis [32], similarly, showed no inhibition of CHIKV infection in SJCRH30 (Fig 3B) and minimal non-dose-dependent inhibition in HSMM (Fig 4B). These results suggest that clathrin-mediated endocytosis is unlikely to be a major entry pathway for CHIKV into muscle cells. To determine the role of caveolae-mediated endocytosis in CHIKV infection, filipin, an inhibitor of caveolae-dependent endocytosis was utilised [33]. Similar to results with monodansylcadaverine and dynasore, filipin treatment did not result in CHIKV inhibition in SJCRH30 cells, and minimal inhibition in HSMM cells (Fig 4C), suggesting that the possibility of caveolae-mediated endocytosis may not be a major pathway involved in the infectious entry of CHIKV into SJCRH30 and HSMM.

**Fig 5:**
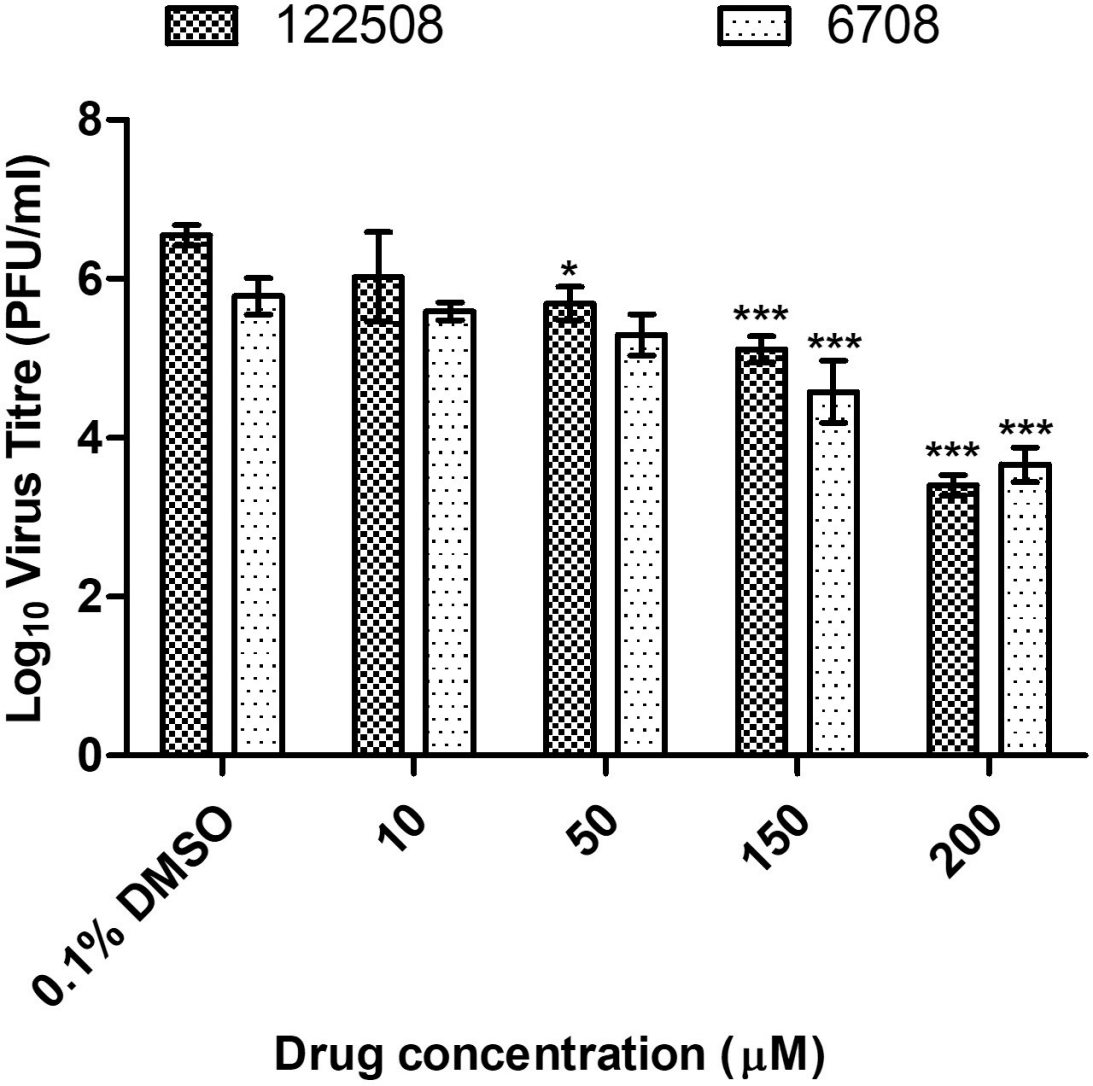
Pre-infection treatment assays of SJCRH30 against CHIKV-6708 and CHIKV-122508 with EIPA. SJCRH30 were pre-treated with EIPA for 3 hours before infection with CHIKV-122508 and CHIKV-6708. Supernatants were harvested at 24 h.p.i for viral plaque assays. The virus titre expressed as log_10_ PFU/mLis plotted against the concentrations of drug used. Significant dose-dependent inhibition of CHIKV-122508 (black-dotted bars) and CHIKV-6708 (white-dotted bars) was observed upon treatment. The asterisk indicates *p values <0.05, **p values of <0.01 and ***p values <0.001.

EIPA, a specific inhibitor of macropinocytosis, was used to investigate the role of macropinocytosis in mediating CHIKV infectious entry [17]. As observed in Fig 3D, a dose-dependent reduction in CHIKV titre was observed upon EIPA pre-infection treatment in SJCRH30, with a 2-log_10_ unit PFU/mlL reduction of CHIKV titre at 200 μM. A similar trend was also observed in HSMM, with a significant dose-dependent reduction in CHIKV titre and approximately 2-log_10_ unit PFU/mL reduction at 200 μM (Fig 4D). These results strongly suggest that macropinocytosis is a major entry pathway for CHIKV infection into both muscle cell lines. Interestingly, a significant decrease in CHIKV titres was observed at a EIPA concentrations of 10 μM onwards in SJCRH30 cells, while HSMM cells showed a significant decrease in CHIKV titres at EIPA concentrations of 50 μM onwards. The EIPA treatment was repeated with the other two CHIKV strains, CHIKV-122508 and CHIKV-6708 in SJCRH30. Confirming data from earlier experiments, results showed significant dose-dependent inhibition of 2-log_10_ PFU/mL reductions at EIPA concentrations of (Fig 5), strengthening the role of macropinocytosis as an important entry pathway in CHIKV muscle cells.

### EIPA treatment in other susceptible cell lines

**Fig 6:**
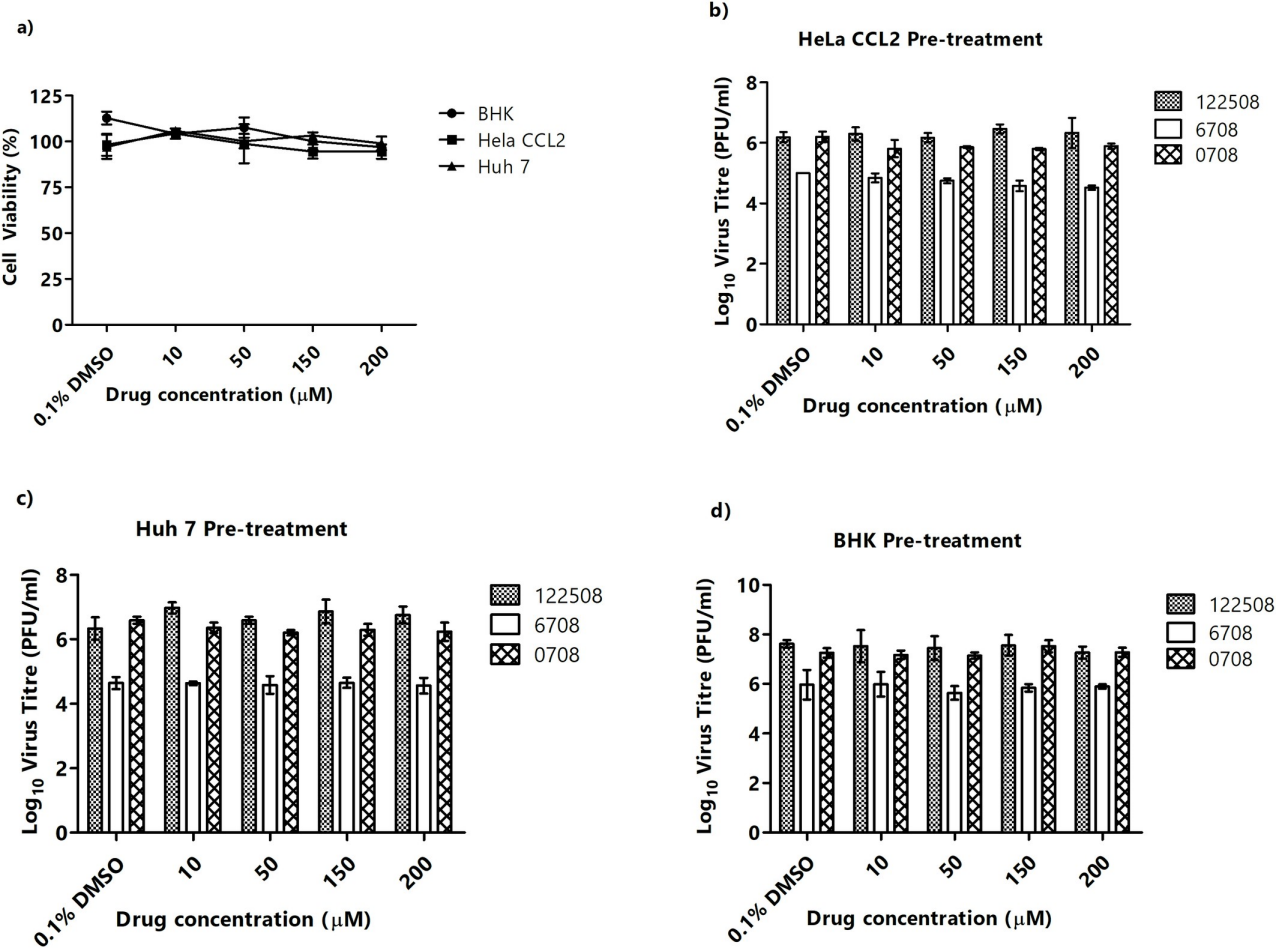
5-EIPA treatment in other susceptible cell lines. (A) Cell viability assay was carried to ensure EIPA treatment was not cytotoxic to cells. Selected cell lines, (B) HeLa CCL2, (C) Huh 7 and (D) BHK-21 were pre- treated with EIPA for 3 hours and infected with three different CHIKV strains, CHIKV-122508 (black bars), CHIKV-6708 (white bars) and CHIKV-0708 (striped bars). Minimal inhibition of CHIKV infectious entry was observed. The asterisk indicates *p values <0.05, **p values of <0.01 and ***p values <0.001.

To investigate the role of macropinocytosis in non-muscle cell lines, EIPA pre-infection treatment was also carried out in with HeLa CCL2, Huh 7, and BHK-21. Prior to infection, a cell viability assay was carried out, revealing minimal toxicity of EIPA in all cell lines tested (Fig 6A).

Upon pre-infection treatment with EIPA and infection with three different CHIKV strains (CHIKV-0708, CHIKV-6708 and CHIKV-122508), minimal inhibition of virus titers was observed across all three cell lines (Fig 6A–6D), suggesting that the entry process of CHIKV into these non-muscle cell lines are unlikely to involve the process of macropinocytosis.

### siRNA-mediated gene knockdown of SNX9 in SJCRH30

To further validate the involvement of macropinocytosis in mediating the infectious entry of CHIKV into SJCRH30, siRNA-mediated knockdown of SNX9 was carried out. SNX9 belongs to the sorting nexin family of proteins, involved in intracellular trafficking, endosomal sorting and early-stage macropinosome formation [34–36]. Minimal cytoxicity was observed upon gene knockdown of SNX9 and non-targeting controls in SJCRH30 (S3 Fig).

The effective knockdown of SNX9 was first validated by Western blot analysis. Dose-dependent reduction in SNX9 protein expression level was observed when compared to non-targeting siRNA controls, showing that gene knockdown of SNX9 was effective (S2 Fig).

**Fig 7:**
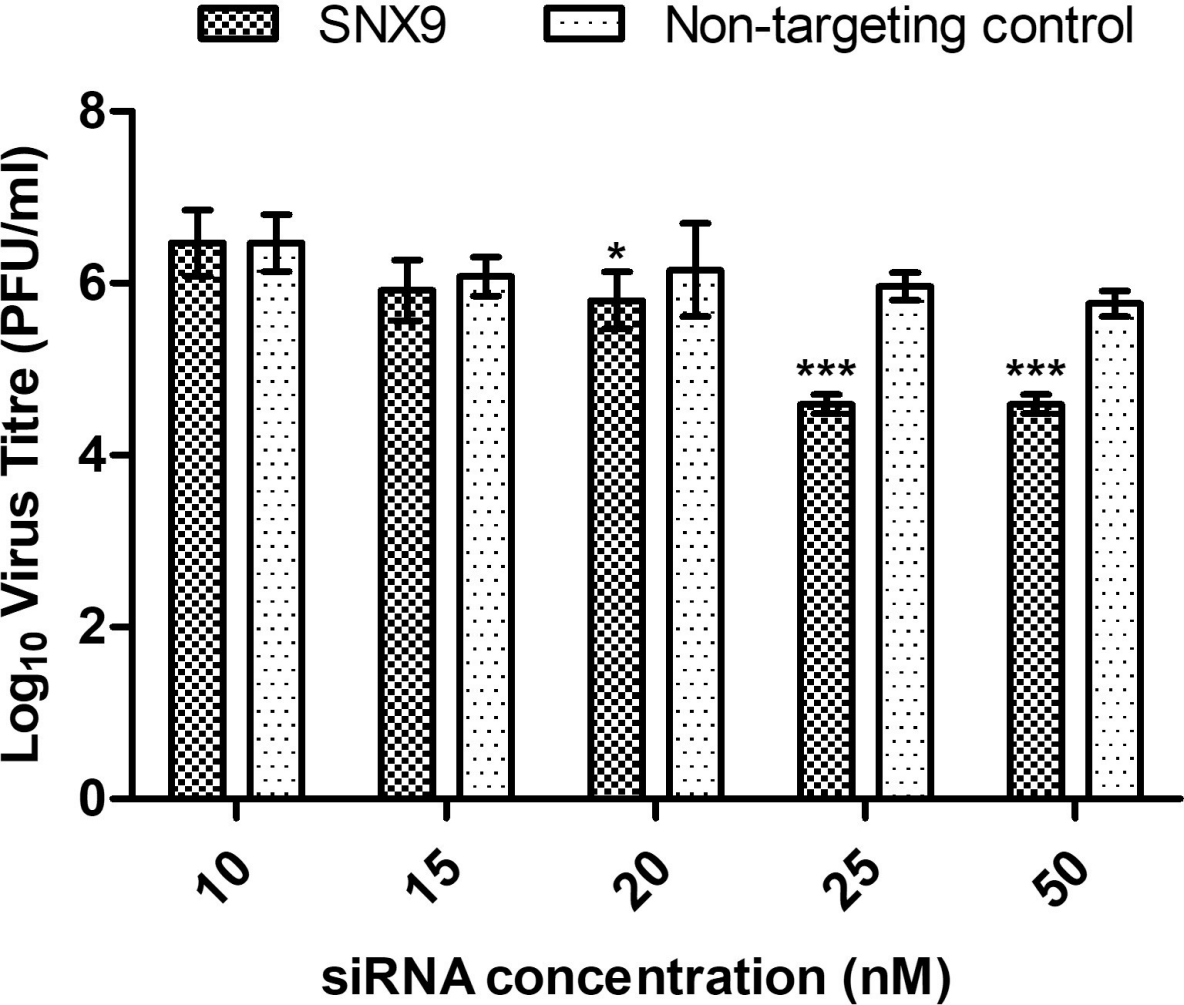
SNX9 gene knockdown showed significant dose-dependent inhibition. siRNA against SNX9 was transfected into SJCRH30 at different concentrations (10–50 nM) for 48h prior to CHIKV-0708 infection. Significant dose-dependent inhibition of CHIKV infection was observed from 25 nM to 50 nM, with approximately 2-log_10_ PFU/mL reduction in virus titre. The asterisk indicates *p values <0.05, **p values of <0.01 and ***p values <0.001.

Upon siRNA-mediated knockdown of SNX9 in SJCRH30, a significant dose-dependent inhibition of CHIKV titre was observed (black bars) concentrations when compared against non-targeting siRNA controls (white bars). Maximal inhibition was observed at 25nM and 50nM concentrations, which resulted in a significant 2-log_10_ inhibition of CHIKV titre, strongly suggesting the importance of SNX9 in the macropinocytosis of CHIKV infection (Fig 7).

### EIPA inhibits uptake of FITC-dextran of SJCRH30 cells infected with CHIKV-0708 strain

**Fig 8:**
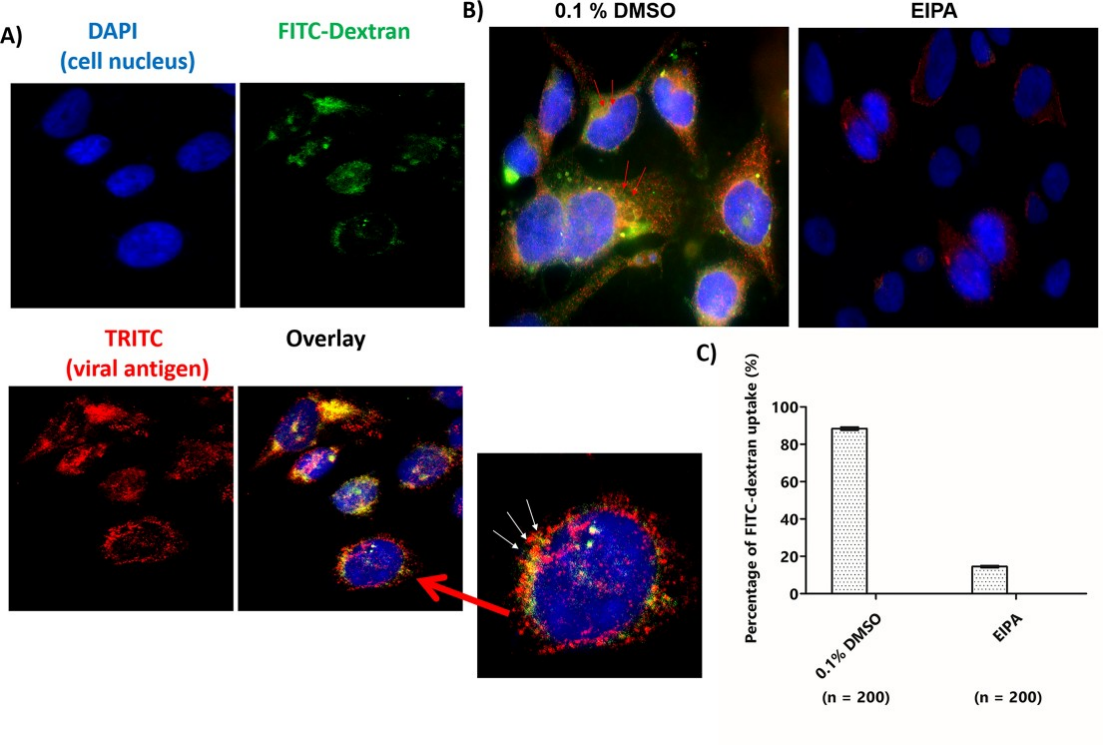
EIPA inhibited uptake of FITC-dextran of SJCRH30 cells infected with CHIKV-0708 strain. SJCRH30 cells were incubated with CHIKV-0708 at 4°C for 30 mins to facilitate virus binding and shifted to 37°C for 10 mins for viral entry. FITC-dextran were added at 37°C for 30 mins and fixation was carried out. The cells were then processed for immunofluorescence assay. (A) Colocalisation (yellow) was observed between CHIKV particles (red) and macropinosomes (green) in the enlarged image (white arrows). The cell nuclei are shown in blue. (B) CHIKV-0708 inhibition upon 0.1% DMSO and EIPA treatment. The cells were pre-treated for 2 h before subjected to CHIKV-0708 infection. FITC-dextran were added for 30 mins and processed for immunofluorescence assay after fixation. Colocalisation (yellow) was observed between CHIKV particles (red) and macropinosomes (green) in 0.1% DMSO-treated cells (red arrows), while no dextran uptake was observed in EIPA-treated SJCR30 cells. (C) Quantitation of FITC-dextran uptake in the presence and absence of EIPA treatment. A total number of 200 cells were counted and expressed as percentage comparing to 0.1% DMSO.

FITC-dextran is well-known to be a fluid-phase marker for tracking of macropinosomes [28,37]. To further characterize the involvement of macropinosomes in CHIKV infection, FITC-dextran was used to track macropinosomes during entry of CHIKV particles into SJCRH30 cells at 30 mins post-infection. Results indicated a strong co-localization between CHIKV particles (red) and FITC-dextran (green) surrounding the perinuclear regions of the cell (yellow, indicated by white arrows) (Fig 8A).

Previous studies showed that EIPA was well-known to inhibit dextran uptake upon virus infection [9,38]. Therefore, SJCRH30 cells were pre-treated with EIPA (200 μM) and subjected to CHIKV-0708 infection at M.O.I 10. Results showed that EIPA inhibited the uptake of FITC dextran upon CHIKV-0708 infection, comparing to the 0.1% DMSO control, where co-localization of between CHIKV particles (red) and FITC-dextran (green) is seen in the absent of EIPA (Fig 8B), agreeing with data from previous figures and providing further proof that that CHIKV utilises macropinocytosis as a major entry pathway into SJCRH30. Additionally, the quantification of FITC-dextran and CHIKV-0708 particles uptake into cells in the presence or absence of EIPA treatment are shown in Fig 8C.

## Discussion

In this study, the susceptibility of eighteen cell lines to CHIKV infection was evaluated using studies of viral growth kinetics. While most of these cell lines have been previously shown to be either susceptible or non-susceptible to CHIKV infection, several of these cell lines were tested for permissiveness to CHIKV for the first time. Cell lines such as SJCRH30, SK-N-SH and C2C12 have not been previously evaluated for their susceptibility to CHIKV infection. Indeed, these cell lines were highly permissive in supporting the replication of all three virus strains SJCRH30 and C2C12 cells are derived from muscle tissues and are fibroblastic and myoblastic in nature, respectively. Their high permissibility to CHIKV infection is hardly surprising, given the known tropism of CHIKV, which includes fibroblasts and myoblasts *in vivo* [39,40]. Results from IFA showed the accumulation of envelope proteins within the cytoplasm and along the plasma membrane, which is the site of virus release via budding, confirming previous reports on alphaviral egress [25]. In contrast, there were two interesting cell lines, namely Huh 7 and HepG2, showed to be permissive to only CHIKV-122508 and not the other two virus strains. This could likely be due to the differences in the strains and the passaging of the viruses in different cell lines.

The elucidation of virus entry processes into host cells has always been of great importance in contributing to the discovery of anti-viral drugs targeting the early process of virus replication. Many previous studies have shown that numerous viruses enter via the common pathways such as receptor-mediated and/or clathrin-mediated pathways [30,41]. While the involvement of clathrin-dependent endocytic pathways in the entry of *alphaviruses* like SINV and SFV has been extensively studied [16,18,36,42], the role of macropinocytosis in mediating the entry of *alphaviruses* has not been explored. Many studies have shown that EIPA, a specific drug inhibitior of macropinocytosis, where it showed to block the activity of Na(+)/H(+) exchangers, which are plasma membrane proteins implicated in all forms of macropinocytosis [12,43,44]. A recent study on human carcinoma cell showed that EIPA can block the activation of the Rac1 and Cdc42 signaling pathways by lowering submembranous pH, therefore, macropinocytosis pathway was inhibited [44]. Previous study on Ebola virus infection also showed that the matrix protein VP40 used macropinocytosis and clathrin-mediated endocytosis to enter cells. It was further supported with the assay of FITC-dextran uptake indicating macropinocytosis as the main entry mechanism and was further confirmed with EIPA drug-treatment [37]. Additionally, study on herpes simplex virus (HSV)-1 gene expression and internalization showed reduction upon EIPA treatment in Vero and HeLa cells [43]. These studies showed that EIPA is effective in inhibiting different viruses in different cell lines. To further validate the involvement of macropinocytosis in mediating the infectious entry of CHIKV into SJCRH30, sorting-nexins 9 (SNX9) gene was selectively knockdown using siRNA gene silencing technology, SNX9 belongs to the sorting-nexins family, where they are involved in intracellular trafficking and endosomal sorting. Previous study on HEKFlp-In cell have shown that SNX9 to associated with the formation of early-stage macropinosome [45].

Given the high permissiveness of SJCRH30 cells to CHIKV infection in this study, as well as the importance of muscle tissues in the *in vivo* pathogenesis of CHIKV infection, we explored the endocytic pathways mediating entry in SJCRH30 and HSMM cells. Pre-infection treatment with inhibitors of clathrin and caveolae-endocytosis resulted in minimal and/or non-dose dependent inhibition of CHIKV infection. Conversely, pre-infection treatment with EIPA, an inhibitor of macropinocytosis, was found to result significant reductions in CHIKV infection, thus indicating that macropinocytosis may play a major role CHIKV entry into muscle cells. siRNA-mediated knockdown of SNX9 also significantly reduced CHIKV titres in a dose-dependent manner and FITC-dextran was found to be inhibited upon EIPA-treated SJCRH30 cells, further confirming the importance of macropinocytosis to CHIKV infection in muscle cells.

Taken together, this study showed for the first time that CHIKV utilized macropinocytosis to mediate infectious entry into human muscles cells. The importance and implications of this mode of infectious entry on infection of muscle cells by CHIKV warrants further exploration in suitable *in vivo* models and this may also reveal novel therapeutic targets that can be utilized for development of antivirals against CHIKV infection.

## Supporting information

S1 Fig Susceptibility of twelve different cell lines infected with three Chikungunya strains–CHIKV-122508, CHIKV-6708 and CHIKV-0708. Cells were infected with different CHIKV strains across various timepoints, where (A) HBMEC, (B) Huh 7, (C) Huh 7.5, (D) HepG2, (E) HEK293A, (F) HEK293T, (G) RD, (H) A549, (I) HaCaT, (J) Vero, (K) Vero C1008 and (L) CHO. CHIKV strains CHIKV-0708, CHIKV-6708 and CHIKV-122508 are represented by (black circle), (black square) and (black triangle).
(TIF)

S2 Fig siRNA-mediated knockdown of SNX9. (A) The efficiency of siRNA-mediated SNX9 knockdown was determined using Western blot, with ß-actin as the loading control. A dose-dependent decrease in SNX9 protein levels was observed upon siRNA treatment. (B) The bands intensities of SNX9 were normalised against the ß-actin loading controls and plotted as percentage knockdown when compared against non-targeting controls.
(TIF)

S3 Fig Cell viability of siRNA-mediated knockdown of SNX9 and non-targeting controls. (A) The cell viability of the siRNA-mediated SNX9 knockdown and non-targeting controls were analysed using alamarBlue assay. SNX9 are represented by (black triangle) and non-targeting controls are represented by (black circle).
(TIF)
