# Supplementary figures and images for: Macropinocytosis dependent entry of Chikungunya virus into human muscle cells
Source: PLoS Negl Trop Dis. 2019 Aug 26;13(8):e0007610. doi: 10.1371/journal.pntd.0007610 (PMC6730948; doi:10.1371/journal.pntd.0007610)

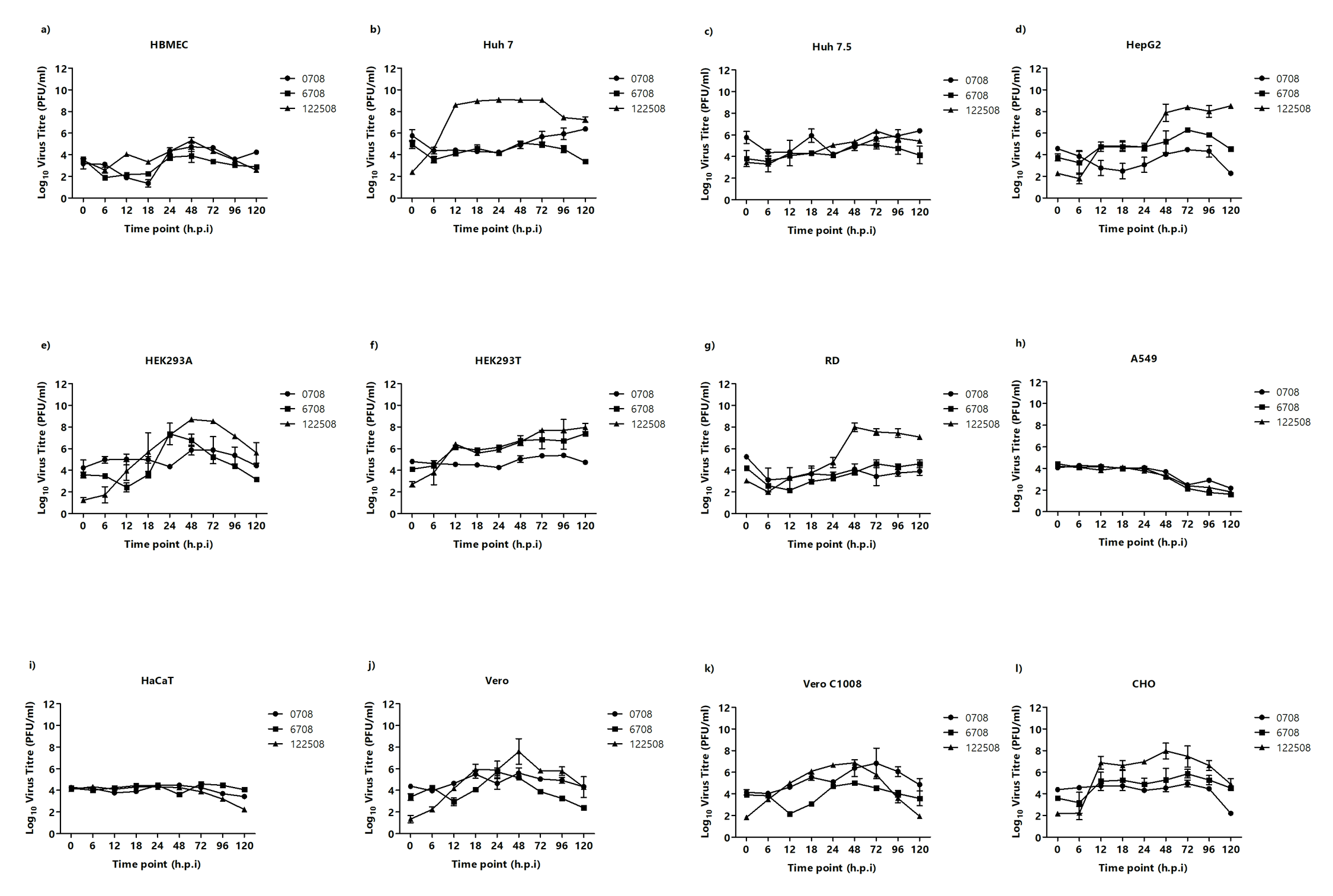

Supplement: S1 Fig — Cells were infected with different CHIKV strains across various timepoints, where (A) HBMEC, (B) Huh 7, (C) Huh 7.5, (D) HepG2, (E) HEK293A, (F) HEK293T, (G) RD, (H) A549, (I) HaCaT, (J) Vero, (K) Vero C1008 and (L) CHO. CHIKV strains CHIKV-0708, CHIKV-6708 and CHIKV-122508 are represented by (black circle), (black square) and (black triangle). (TIF) [file pntd.0007610.s001.tif]

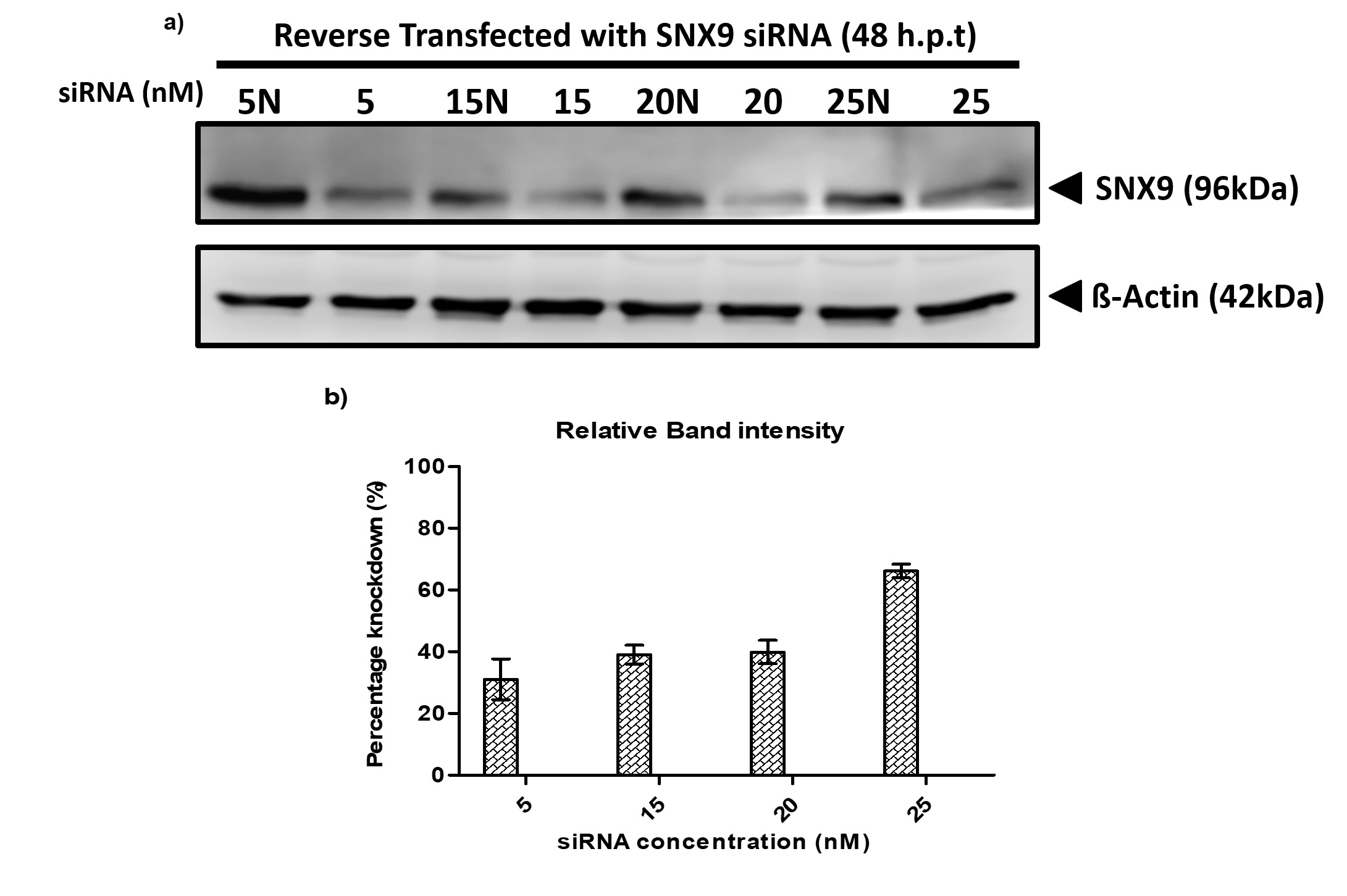

Supplement: S2 Fig — (A) The efficiency of siRNA-mediated SNX9 knockdown was determined using Western blot, with ß-actin as the loading control. A dose-dependent decrease in SNX9 protein levels was observed upon siRNA treatment. (B) The bands intensities of SNX9 were normalised against the ß-actin loading controls and plotted as percentage knockdown when compared against non-targeting controls. (TIF) [file pntd.0007610.s002.tif]

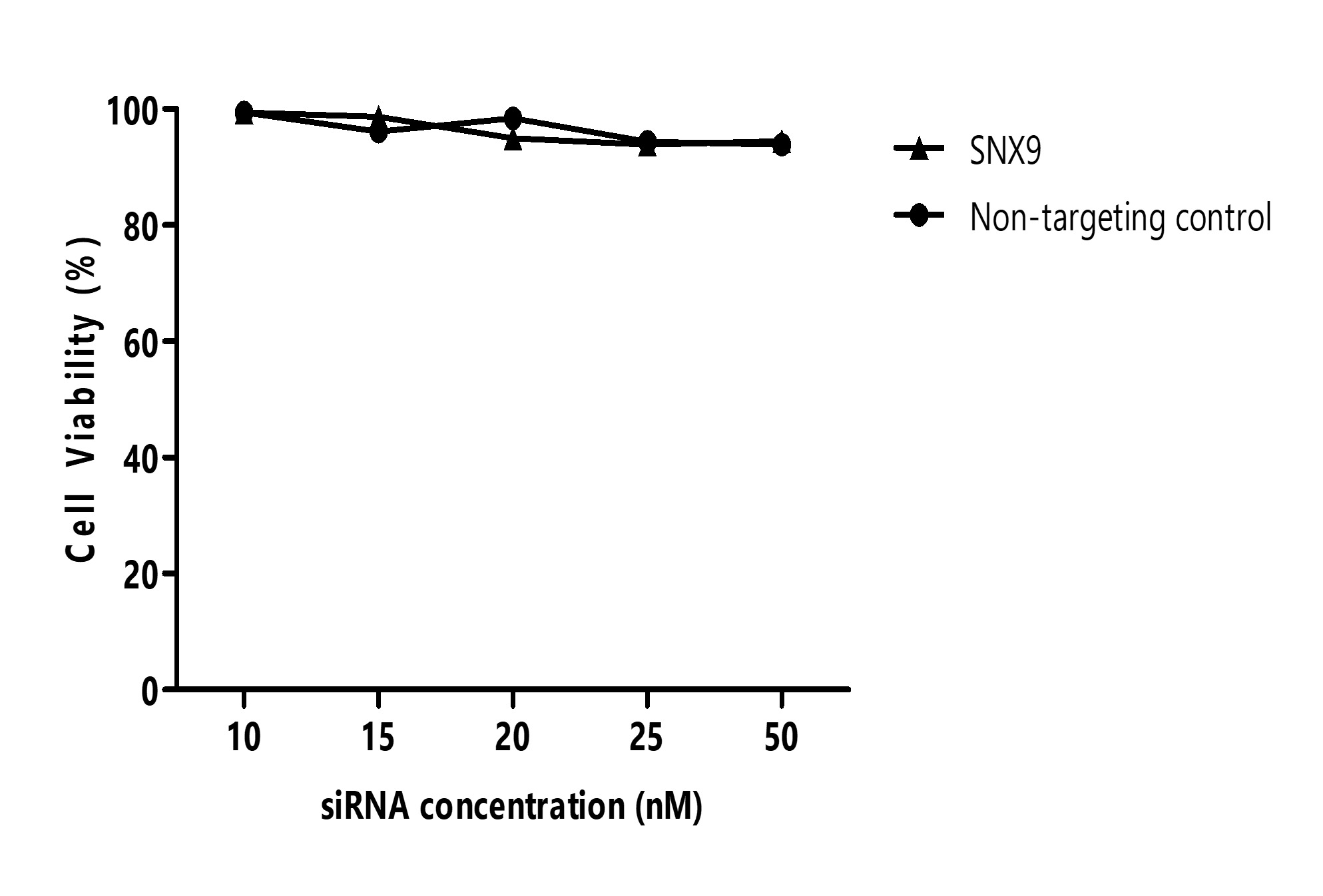

Supplement: S3 Fig — (A) The cell viability of the siRNA-mediated SNX9 knockdown and non-targeting controls were analysed using alamarBlue assay. SNX9 are represented by (black triangle) and non-targeting controls are represented by (black circle). (TIF) [file pntd.0007610.s003.tif]
